# Sowing Seeds for Healthier Diets: Children’s Perspectives on School Gardening

**DOI:** 10.3390/ijerph14070688

**Published:** 2017-06-25

**Authors:** Edris Nury, Asia Sarti, Coosje Dijkstra, Jacob C. Seidell, Christine Dedding

**Affiliations:** 1 Department of Palliative Care, University Medical Center Freiburg, 79106 Freiburg, Germany; 2 Athena Institute for Research on Innovation and Communication in Health and Life Sciences, Amsterdam Public Health research institute, VU University, 1081 HV Amsterdam, The Netherlands; a.sarti@vu.nl (A.S.); c.dedding@vu.nl (C.D.); 3 Department of Health Sciences, Amsterdam Public Health research institute, and the EMGO Institute for Health and Care Research, VU University, 1081 HV Amsterdam, The Netherlands; coosje.dijkstra@vu.nl (C.D.); j.c.seidell@vu.nl (J.C.S.)

**Keywords:** school gardening, health promotion, the child’s perspective, participant observation, vegetables

## Abstract

School gardening programmes are among the most promising interventions to improve children’s vegetable intake. Yet, low vegetable intake among children remains a persistent public health challenge. This study aimed to explore children’s perspectives, experiences, and motivations concerning school gardening in order to better understand and increase its potential for health promotion. Using participant observation and semi-structured interviews, we provided 45 primary schoolchildren (9–10 years) from Amsterdam, who participated in a comprehensive year-round school gardening programme, the opportunity to share their experiences and ideas on school gardening. Children particularly expressed enjoyment of the outdoor gardening portion of the programme as it enabled them to be physically active and independently nurture their gardens. Harvesting was the children’s favourite activity, followed by planting and sowing. In contrast, insufficient gardening time and long explanations or instructions were especially disliked. Experiencing fun and enjoyment appeared to play a vital role in children’s motivation to actively participate. Children’s suggestions for programme improvements included more autonomy and opportunities for experimentation, and competition elements to increase fun and variety. Our results indicate that gaining insight into children’s perspectives allows matching school gardening programmes more to children’s wishes and expectations, thereby potentially enhancing their intrinsic motivation for gardening and vegetable consumption.

## 1. Introduction

Low vegetable intake among children remains a challenging public health concern in both high-income and low-income countries [1]. Children from families with low socioeconomic status (SES) are at a higher risk of unhealthy dietary habits and insufficient vegetable intake [2,3]. School gardening programmes are considered to be a promising intervention to improve children’s vegetable intake as multiple studies have shown that school gardening improves children’s vegetable knowledge and preferences, increases their willingness to taste vegetables, and positively changes their attitudes towards vegetable consumption [4,5,6,7,8]. These studies further suggest that school gardening may, therefore, also increase children’s vegetable intake. For example, a study by Evans and colleagues [9] has found that children with previous school gardening experience did not only have greater vegetable exposure and more diverse vegetable preferences but also consumed more vegetables than children with less gardening experience. Other studies have also suggested positive effects on children’s vegetable knowledge, attitude, and willingness to try vegetables, but found no clear evidence of an increase in children’s vegetable intake or vegetable preferences after participation in gardening or nutrition education programmes with a gardening component [10,11,12,13,14]. 

Although existing findings on school gardening and its effects on children’s vegetable consumption are encouraging, previous studies did not investigate conditions for success from the perspective of children themselves. Designing, implementing, and evaluating effective interventions requires obtaining contextual information about an intervention and its users, as well as information from the perspective of the users [15]. Gaining insight into the user’s perspective allows for a comprehensive understanding of an intervention, identifying strengths, as well as possible opportunities for improvement. Furthermore, it helps us understand what motivates children to participate in school gardening in the first place. Investigating children’s ideas on the purpose of school gardening and comparing them with formal programme objectives may additionally aid in bringing the two more in line with each other, thereby enhancing programme effectiveness.

In Amsterdam, school gardening has existed for more than a century, with its goals evolving over time from school gardening as a solution for major food shortages during and after World War I to gardening as an educational tool for health and food [16]. There are 13 school gardening complexes in Amsterdam where approximately 6800 children participate in a year-round school gardening programme each year [17]. When the first school garden complex was constructed in Amsterdam in 1920, the main goal was to provide city children with an opportunity to interact with nature. Other goals included teaching children how to grow vegetables and getting them interested in nature and agriculture. Later on, more emphasis was put on nature and food education, and gardening during school hours became integrated in most primary schools in Amsterdam [16]. The current Amsterdam school gardening programme does not only aim to teach children about the environment and nature, but also introduces children to food production and educates them on where their food comes from.

The aim of our study was to explore children’s experiences, perspectives, and motivation concerning school gardening and vegetable consumption to better understand and improve its potential for health promotion. In order to achieve this objective we interacted with the children during the entirety of the Amsterdam school gardening programme, observed their attitudes and behaviours towards gardening and vegetables and conducted formal interviews, as well as informal conversations.

## 2. Materials and Methods

### 2.1. Study Design

We chose an ethnographic approach, combining participant observation, formal interviews, and informal conversations. In order to gain a more complete and in-depth understanding of children’s perspectives and experiences, participant observation was chosen as the main ethnographic data collection method. It allowed us to get alongside children in their ‘natural’ environment to explore how they made sense of the world and how they shaped their realities. Ethnography aims to describe and understand social interactions, behaviours, and perceptions that occur within a group of people or culture [18]. Over the years it has been recognized as a fundamental research method to explore and make sense of the social worlds of children [19]. Since ethnography focuses on the perspective of those being studied, e.g., intervention users, it has been recognized as an important part of implementation research in nutrition, helping to understand how delivery and utilisation processes affect programme impacts or outcomes and, thus, how to optimally tailor intervention design and implementation to different sociocultural and environmental contexts [15].

This study was part of a larger mixed-methods study which further consisted of a quantitative survey, aimed at examining vegetable preferences, knowledge, and intake of disadvantaged primary school children in Amsterdam. The current study investigates the year-round Amsterdam school gardening programme which is part of a comprehensive nutrition education programme called Voedselwijs (Foodwise). Other elements of Voedselwijs include farming education, cooking workshops, and a ‘no-waste’ lunch. The Amsterdam school gardening programme consists of 25 structured lessons of 90 min, in which children grow and process vegetables, herbs, and flowers. The first three sessions comprise indoor lessons in which children are introduced to their school garden, study soil types, and learn how seeds grow into plants. In April, weekly gardening activities start during which each child receives a small patch of land to grow plants and vegetables with the help and instructions from a garden educator. After the summer holiday, children harvest vegetables to take home and learn how to make vegetable soup and pizzas using their own vegetables. Furthermore, children pick their self-grown flowers and learn how to arrange them. Finally, the programme ends with an indoor lesson on winter plants and animals, and a food quiz in December [18]. Our study took place from January to December 2015.

### 2.2. Ethics

The study was conducted in accordance with the Declaration of Helsinki, and the protocol was approved on 28 January 2015 by the Medical Ethics Committee (METc) of the VU University Medical Centre (protocol 15/026). A passive parental informed consent strategy was chosen as the study did not involve sensitive topics and it was expected that children would not be exposed to any significant risks. Furthermore, throughout the study, it was emphasized that participation was voluntary and that children were free to withdraw from the study at any moment without having to provide any reasons and without facing any consequences.

### 2.3. Participants

The recruitment process started by inviting primary schools, located in three socioeconomically-disadvantaged areas of Amsterdam which are characterized by a high prevalence of overweight and obesity, to participate in the quantitative study. Schools were invited with the help of the Amsterdam Nature and Environmental Education Centre (ANMEC) which held the contact information of all schools that participate in the school gardening programme of Amsterdam. Schools received an email from ANMEC with information about the study and were asked to participate. Subsequently, schools were given a week to respond, after which those that did not respond were invited again by telephone. A total of 12 schools decided to participate in the quantitative study. Of these schools, two schools were then selected to participate in the current qualitative study using a combination of convenience and purposive sampling. Purposeful sampling was used to identify and select schools that had been participating in the Amsterdam school gardening programme for many years and that were located in city districts inhabited by the population of interest, i.e., children living in low-SES and high overweight and obesity contexts. In this manner, we aimed to include schools that were familiar with the Amsterdam school gardening programme and which were available and willing to participate in our qualitative study.

### 2.4. Data Collection Procedures

Participant observation was used to determine how the school gardening programme was introduced to and received by the children. Participant observation was chosen because it helps the researcher gain insights into the perspectives and actions of the individuals being studied, i.e., in what persons say and do in their ‘natural’ environment [19,20,21]. 

Between March and November 2015, all indoor and outdoor lessons of the children in the two target classes were observed. Two researchers followed children participating in gardening activities, held conversations with them and closely observed their actions and behaviour. Additionally, at the school garden complex of one of the participating schools, one of the participant observers also received a garden patch in between those of the children. This further facilitated observations as it provided a good opportunity to occasionally retreat from being a mere observer and to participate hands-on in the gardening activities, gaining a richer understanding of children’s gardening experiences. Participating in children’s gardening activities also enabled the researchers to gain children’s trust and build rapport. All observations were recorded in the Dutch language using field notes which were expanded into thick descriptions of what was observed, directly after each observation. Thick descriptions do not merely present the actions of participants but more detailed information about context, emotion, and the different relationships between participants [22].

Observations further provided valuable input for interviews and informal conversations with the children. Informal conversations occurred spontaneously during gardening activities and were open-ended, enabling the researchers to establish rapport with the participants and to get to know their thoughts on gardening.

In addition, a total of 22 semi-structured interviews were conducted, 18 with the children, two with the school teachers and two with the garden educators. All interviewees granted permission to audio-record interviews, after which the interviews were transcribed verbatim and anonymised using pseudonyms. Both interviews and informal conversations were conducted in Dutch.

Semi-structured interviews with the children took place at school and lasted between 20 and 30 min. Children were interviewed in locations outside the classroom which the children had chosen themselves and where they felt comfortable. School teachers and garden educators were interviewed for contextual information about how children reacted to programme elements and what elements were, from their experience, liked or disliked by children.

### 2.5. Data Analysis

Data analysis was an iterative process and started directly from the first meeting at the school gardens, i.e., when raw field notes were expanded into narratives, elaborating on initial observations, and when interviews were transcribed. Transcribed interviews and expanded field notes were analysed using qualitative content analysis in an inductive manner, i.e., themes ‘emerge’ from the data through a process of open coding and theme refinement, without restricting the analysis by predefined codes and themes [23]. This process took place in three steps: open coding, categorization, and abstraction [24]. The content analysis started by coding eight interviews and three field reports by hand using open coding, prior to coding all collected data in the MAXQDA 10.4 data analysis software programme (VERBI GmbH, Berlin, Germany). Open coding involved reading and reviewing the transcripts multiple times, while writing notes and headings, i.e., codes, in the manuscript to describe all content. Recurring or striking patterns and apparent inconsistencies between children’s beliefs and their actions were identified and coded. After repeated reviewing, all codes were transcribed onto a coding sheet, forming categories. Relationships between different data segments were explored and both recurring categories and categories describing similar thoughts or events were grouped into broader higher order categories to reduce the number of required categories. Negative cases, i.e., views and events that contradicted or did not support major patterns and explanations that were emerging from data analysis [25], were identified to obtain a more complete and in-depth understanding of children’s experiences and perspectives. Finally, categorization allowed initiating the process of abstraction of the data, i.e., developing general descriptions of the research subject by moving from the specific and anecdotal to the general [23]. In this manner higher order categories were grouped into subcategories (or subthemes), which in turn were grouped into main categories (or main themes). Both coding sheet and interim results of the data analysis were discussed in regular group meetings with the research team.

Since all data was collected in the Dutch language, the data analysis was performed in Dutch. During data analysis and write up of the manuscript, the original Dutch quotes were used for as long as possible to prevent losing meaning as a result of translation. The quotes in the final manuscript were translated by the authors and checked by a native English speaker who also speaks Dutch.

## 3. Results

A total of 45 children participated in the study, 18 boys and 27 girls, while three children were opted out by their parents. Children were nine or 10 years old at the start of our study, reflecting the target population of the school gardening programme. Participants were all born and raised in the Netherlands, but have diverse ethnic backgrounds: Dutch (*n =* 27), Moroccan (*n =* 5), Surinamese (*n =* 2), and Afghan (*n =* 1). In addition, some children were of mixed Dutch-immigrant descent (*n =* 6), mixed non-Dutch descent (*n =* 1), and of unknown descent (*n =* 3). Table 1 presents the participant demographics. 

First, we describe children’s general impression of the programme, followed by what they consider to be the purpose of school gardening. We conclude with children’s own ideas for improvements to the current programme.

### 3.1. Children’s General Impressions of School Gardening

Children enjoyed the school gardening programme. As one boy put it: “Well, I just think it’s really fun, school gardens and even garden work” (Stan). Children stated that the programme was fun for a number of reasons, such as being active outside and doing something fun during school time but, in particular, because it enabled them to learn a variety of new things in an independent, playful, and fun way. As Kees told us: “There are lots of things to do and we also learn a lot.” Theo expressed: “I really like it because you have your own garden. So, people will not meddle with you. For example, if you’re doing something wrong, you can learn from it.” Being away from school during school time was by and large appreciated by the children, as illustrated by Rob: “I don’t like being in school in the afternoon. So I think it’s nice that there are things to do in the school garden.” Witnessing the growth of your own crops and harvesting them was another often heard reason for liking the programme. Although the majority of participants was very positive about school gardening, one girl said that while she did believe that the programme was nice, gardening just was not for her: “It’s nice, but sometimes I find it somewhat less fun because gardening is not really my thing. Ehm, I like doing sports things, and not really things with plants and vegetables. And making holes in the soil and so on…with worms and stuff” (Vanessa).

Whilst a minority mentioned that the indoor classroom lessons were also fun and informative, all agreed that the outdoor gardening lessons were far more enjoyable. During the indoor lessons, it was observed that children were frequently bored, did not know what to do when they had finished their workbook assignments or just lost interest, as illustrated by Sander: “[…] we had to glue seeds in the workbook, glue stuff in it and colour it in and write. Well, I did not feel like doing all that. I was thinking ‘just let me go and dig in the mud outside.’” In contrast to this, outdoor gardening lessons often started with children cheerfully entering the school garden complex and curiously and enthusiastically running to their gardens. Furthermore, when garden educators announced outdoor lesson cancellations due to holidays, most children were visibly disappointed. Some children even asked whether it was possible to reschedule cancelled lessons. Children stated that they preferred the outdoor gardening lessons because they could do more themselves and because it allowed them to actively and independently perform hands-on activities outside: “You do more than during the indoor lessons. Because then you just sit and learn everything and you have to answer questions. But if you do it outside, you really have to do it yourself. So I think it’s more fun” (Theo). Moreover, whereas children were initially having difficulties with gardening and required much help from gardening educators, after several gardening lessons they were capable to confidently take care of their gardens on their own, visibly enjoying gardening activities.

A majority of the children described harvesting as the most enjoyable school gardening activity, closely followed by planting and sowing. Participants stated that they liked harvesting because they could take their self-grown vegetables home and eat them. For example, Tim said: “And I like harvesting the most, of course. So that you have tasty food. […] that you can just enjoy eating your own food.” Likewise, Vanessa commented: “Harvesting because then you can have yummy fresh food once a week.” Elaborating on this point, many children said that it is also “more fun to eat your own food.” Children’s eagerness for harvesting was evident starting from the first outdoor gardening lesson, when it was observed that children would regularly and impatiently ask the garden educator when harvesting would start. When the children finally harvested for the first time, they were overjoyed. It started at the sitting corner, when a girl fanatically started yelling “Harvesting people! It says harvesting” when she noticed a word web about harvesting. The news then spread like a wildfire as more and more children started cheering and drumming on their watering buckets. Similar to harvesting, planting and sowing were also liked and linked to food by most children: “Well, I do actually like planting and sowing because you can do things. Then it will grow and then you can sooner make something tasty at home” (Rob). Others stated that they favoured planting and sowing as it was something they rarely did and because they enjoyed these activities: “I just think it [planting] is fun to do because it’s just something else. Because I almost never do it.” (Melisa) However, a minority experienced planting and sowing as less enjoyable because they had difficulties determining where to plant or sow: “Sometimes I think it’s hard. […] Then it don’t know where to plant, but I do pay attention. But I don’t know where…” (Vanessa).

Although children were generally enthusiastic about the school garden lessons, some aspects were disliked. It was well observed that long explanations and instructions at the sitting corner or the demonstration garden often caused impatience and frustration among the children. As Sam commented: “The instructions…I think they are always taking too long.” This made it difficult for the school garden educators to finish giving their instructions as children started talking to each other, playing with their buckets or even wandering to their garden patch while instructions were being given. Children’s discontent with long instructions was exacerbated by the number of explanations and instructions given at a time, making it difficult for children to remember everything. Lana stated: “That you have to remember what to do, otherwise you have to ask all the time. Because sometimes we have to do a lot of things and then it’s hard to remember everything.” 

Additionally, field observations revealed that children did not like it when there was not enough time to complete assignments. Despite children supporting and helping each other with gardening assignments throughout the programme and gardening lessons, there were always some children who were not able to finish all their gardening assignments before the end of each lesson, leaving them worried and dissatisfied when departing from the school garden. As one of the children put it: “[…] it’s just that sometimes we have too little time to do things and I think that’s annoying. I’m just only halfway through and then we have to stop again” (Miranda).

Weeding was experienced as one of the most unpleasant and frustrating activities as children were frequently confused because they found it difficult to distinguish weeds from crops. This sometimes resulted in children accidentally raking or pulling out crop plants, leaving them visibly upset. Similarly, watering plants was also one of the most challenging and disliked tasks, often causing a muddy mess in both the garden and on children’s outfits. For example, Tom said: “Yes, watering actually. Because it then floods a little. […] Yes, it then flooded and then you had to put sand on it. But then your boots became dirty.” Although children were appropriately dressed for gardening, not all of them liked to become dirty.

### 3.2. Children’s Perspectives on the Purpose of the School Gardening Programme

Although children had different ideas about the purpose of school gardening, a clear majority believed that the purpose was just to have fun. As Rob expressed: “I think the goal is....um...just having fun!” Learning how to independently garden, especially how to plant, sow, and harvest was believed to be another important purpose of school gardening. Children believed this to be important because it would enable them to start their own gardens in the future, as illustrated by Rob: “[…] because then I can start my own garden later. […] I think it’s important for later because it’s handy to know how to plant. Then you can also explain it to your children.” Elaborating on this, many children said that they were eager to learn how to garden in order to be able to garden at home and help out parents or relatives with garden work. Similarly, participants who habitually gardened (e.g., at home or with relatives) noted that school gardening could help them improve their gardening skills. As Camilla explained: “And now I can improve myself and then I can help my grandpa even more [with gardening]!” Max added: “I’ve already learned how to handle stuff. For example, how to handle the shovel and rake.”

A small number of participants felt that the purpose of school gardening was to take home vegetables and encourage children to eat more vegetables. As one of them, Tim, put it: “I feel that the purpose of the school gardens is that you eat more vegetables…when you never eat vegetables at home. Then you have grown your own vegetables, and then you just want to eat them yourself. […] So the goal is that you eat more vegetables.” Most participants believed that participation in school gardening could, indeed, have a positive effect on children’s vegetable intake as they felt that children would be eager to eat the vegetables they had put so much effort into. For example, Michelle said: “I would be very proud of a big harvest, with onions and potatoes. I mean, you’ve planted them yourself and then you do want to eat them.” Others believed that participation would make children curious about the taste of their own vegetables, as illustrated by Camilla: “Yes, if they participate they’ll learn that vegetables can also be tasty because they get to know the vegetables. […] I first didn’t like lettuce but because of school gardening I do like it now. Because I thought: ‘ok, I’m going to taste it.’” In contrast, three participants felt that school gardening would not necessarily lead to an increase in the vegetable consumption of children, explaining that they did not believe it would take away children’s dislike for vegetables: “If you don’t like vegetables, I don’t think you’re going to eat more vegetables [because of school gardening]” (Sam).

Finally, a minority of the participants thought that the programme aimed to teach children more about nature and plants, and how to deal with them respectfully, as Rashid told us: “I think the purpose of the school gardening programme is to learn about nature. About plants, mountains, sand...things like that.” Several children elaborated on this point, stating that it would help them distinguish edible plants from non-edible or harmful plants. As Miranda put it: “Then you’ll at least know what a plant is called and what it is. […] Because if you don’t know, you might think: ‘I’ll just put that [plant] in my mouth.’ And then you don’t know, maybe it has prickles or it’s poisonous.” Similarly, some children pointed out that it is important to know what you eat and whether it is treated with pesticides or not. Camilla commented: “I think it’s important because we can’t live without vegetables. This is our food, our vitamins.”

### 3.3. Children on Opportunities for Improvements

Although children were enthusiastic about the programme in general, they did have suggestions for improvements. Almost all children believed that the programme could be improved by taking better measures to facilitate gardening assignments. For example, children suggested to provide more time to finish gardening assignments. As Vanessa commented: “A tip…yes, just a bit shorter explanations so that we have more time to plant and harvest.” Furthermore, many children suggested that garden educators could take better measures to facilitate gardening assignments by, for example, using plant tags more often to reduce confusion on what to plant where; providing children with description cards for the gardening assignments; ensuring that sufficient gardening tools and growing plans are available; and building an additional demonstration garden for children who have their gardens on the opposite side of the complex to reduce confusion on where to plant. 

A majority of the children also wanted additional activities at the school gardens to increase fun and variety. During informal conversations in the school garden, many children mentioned that it would be more fun to have contests on who has the tallest plants and most beautiful vegetables: “Actually, I think it would also be very fun to decide, for example, who has the most beautiful vegetables and to also discuss that in class” (Vanessa). Furthermore, a number of children suggested a scavenger hunt and a plant quiz, involving awarding prizes: “[…] for example, do something like a scavenger hunt. That you then have to search for as many worms as possible in your garden within five minutes. And the one who has the most wins something” (Latifa).

Finally, a number of children would like to see bigger gardens so that more crops could be planted. As Frank put it: “I would make bigger gardens. […] No, I don’t think they’re too small, but you can plant more stuff if you have bigger gardens.” Larger gardens would also better meet children’s demand to let them decide for themselves what to grow, for example strawberries, blackberries, cucumbers and cauliflowers: “That you can just choose what you want to plant […] it would be more fun to just take anything you want and plant as many of it as you want” (Melisa). Similarly, some children stated that it would be fun to have the space to experiment with growing plants by planting or sowing them differently: “Maybe have some kind of experimental garden where you also can plant stuff. Only then do it really differently and see how that works. For example, not putting the seeds in a hole but just putting them on the soil and then scatter soil over them. And then see how that works out” (Sam). It was believed that this would provide children with the opportunity to learn from both successes and failures in a processes of trial-and-error: “[…] that you can try. That you have a couple of chances to do it. So if you then do it the wrong way it doesn’t matter much. Because then you can see for yourself what happens” (Lana).

## 4. Discussion

This study aimed to fill knowledge gaps concerning children’s experiences with and perceptions of school gardening. The small amount of qualitative research on school gardening that does exist has focused on children’s general gardening experiences, mainly reporting positive gardening experiences [26,27,28,29], but none have shed light on children’s ideas of its purposes, their motivations for gardening, and their ideas and suggestions for improvements. By providing children, the primary intervention users, with an opportunity to voice their views on what they believe works and what does not, unique input for programme changes and improvements is gained.

Consistent with previous qualitative studies on children’s gardening experiences [26,27,28,29], our study shows that participants were enthusiastic about school gardening and generally enjoyed it. Similar to findings by Passy, Morris, and Reed [30], children clearly favoured outdoor hands-on gardening activities. Indeed, earlier research has shown that young students prefer a hands-on, inquiry-based learning approach over traditional textbook based methods, and developed more positive attitudes towards schooling and science as a result [31,32]. Harvesting was mentioned as the most enjoyable activity, closely followed by planting and sowing. Children were curious and gained a sense of accomplishment and pride from seeing their own vegetables, growing, harvesting, and taking them home for consumption. These findings are consistent with other studies demonstrating that children experience feelings of achievement, satisfaction and pride from taking care of their crops and harvesting them [29,33]. On the other hand, children specifically disliked having to listen to long gardening instructions and having little time for assignments which, to the best of our knowledge, is not documented in previous studies. Assessing the likes and dislikes of children is relevant because it provides useful insights into what influences children’s motivation to garden.

Our results showed that children’s primary motivation for engaging in the school gardening programme is ‘having fun’, which is consistent with data obtained by Bowker and Tearle [27]. Experiencing enjoyment or fun is a vital component for intrinsic motivation. Whereas extrinsic motivation entails performing activities because of an external demand or reward, intrinsic motivation involves doing something volitionally, mainly because it is enjoyable or fun. Research has shown that individuals with intrinsic motivation toward a certain behaviour show greater effort, commitment, and perseverance in displaying that behaviour [34]. The Self Determination Theory (SDT) of Deci and Ryan [34] argues that nurturing intrinsic motivation requires satisfying three innate psychological needs: autonomy (i.e., the need to feel free to act of your own volition, without others deciding for you), competence (i.e., the need to feel capable and proficient to accomplish desired goals), and relatedness (i.e., the need to feel connected and belonging to your social environment). School gardening appears to provide children with opportunities to experience these three psychological needs. To a certain degree, it offers environments that support children’s autonomy, enabling them to have their individual garden which they can take care of independently without others meddling. Moreover, despite the highly structured programme setup, children did experience some autonomy in gardening, for example, gardening with or without gardening tools. Children’s experiences of autonomy were stimulated most when harvesting as they could not only decide what and how many crops they wanted to take home but were also free to decide what to do with them at home.

In addition, school gardens proved to be excellent ventures to experience feelings of competence as children gained a sense of achievement and pride, resulting from a rigorous, but rewarding, gardening journey. Putting effort into planting, sowing and nurturing crops, witnessing them grow and, finally, harvesting and enjoying the result of their hard work enabled children to feel competent and confident. After several gardening lessons, children were capable of gardening properly without the help of gardening instructors, using gardening tools in a correct and safe manner.

Lastly, school gardening also contributed to feelings of relatedness as children felt connected throughout the programme, consistently helping and supporting each other with gardening activities. These feelings of relatedness probably extended beyond the school gardens as children believed that the programme helped them to support others in their close social environment (e.g., parents, friends, and grandparents) with gardening. In this manner, school gardening has connected them with other gardeners, both young and old. 

When comparing the programme’s formal goals with children’s goals and their ideas on the purpose of school gardening, they seem to match well. Similar to the programme goals, participants believed that the main purposes of school gardening were to teach children how to garden properly, teach them more about nature, plants, and vegetables, and enable them to harvest, take home, and consume fresh vegetables. Almost all participants believed that participation in school gardening would positively influence children’s vegetable intake. Learning more about vegetables, performing gardening activities in a vegetable-promoting environment, harvesting, and taking home self-grown vegetables was believed to motivate children to try new vegetables or consume more vegetables.

Finally, although children had many specific requests and suggestions for improvements, perhaps the most striking one was their request for more opportunity for exploration and experimentation. Children, paradoxically, valued the school gardening programme structure but, at the same time, they desired more autonomy to decide for themselves what to grow and to experiment in their gardens. The importance of experimentation has been described by Jarret [35] (p. 2), a scholar in the field of inquiry strategies for science and mathematics learning, who considers that children “construct their own knowledge by actively taking charge of their learning” and that just providing good teaching and quality textbooks is not sufficient. This is in line with recommendations to arrange education in such a manner that facilitates children to take charge of their learning actively by posing questions, conducting their own experiments, experiencing and learning from both successes and failures, and communicating their findings with others [32,35]. Hart [36], professor and director of the Centre for Human Environments and the Children’s Environments Research Group, describes this as ‘participatory learning’ and stresses the importance of providing children, the learners, with a key role in their own learning process. He argues that children should be taken seriously, have choices, and should be actively involved and consulted in their learning process. When these conditions are met, participatory learning can take place in which children investigate, interact, and learn from each other and adults acquire a valuable opportunity to see and understand how children make sense of their lives and their surroundings. It should, however, be noted that merely providing more opportunity for experimentation or additional activities may not be adequate as sufficient time is also required for children to be able to explore and experiment or perform additional activities in the school gardens.

Limitations of this study lie in the limited numbers of participating schools and children. In addition, although formal interviews were conducted to gain insights into individual children’s ideas, due to already-constrained school timetables it was not possible to interview all children. It could be further argued that the positive results were due to the fact that the participating schools and schoolteachers were the most enthusiastic and willing to participate in our study. This may have influenced children’s attitudes and perceptions in favour of nature, gardening, and vegetables. Therefore, it is important to bear in mind the possible bias in children as positive reported outcomes may not have been solely due to participation in the Amsterdam school gardening programme. Finally, there was a lack of information on if and how long vegetable-promoting effects persisted outside the school gardens and after completion of the programme. Similarly, it was not determined to what extent formal programme goals, namely educating children on the environment, nature, and origins of food, were achieved.

The key strength of this study is the use of participant observation, which proved to be a powerful method to gain insights into children’s actions and perspectives in a natural setting. Using this method we were able to avoid disrupting children’s natural behaviour and could truly focus on their experiences and views. Informal conversations and semi-structured interviews with children complemented observations and provided additional insights into children’s perspectives and behaviours, both inside the school gardens and at school. This resulted in rich data and improved the validity of the results. 

## 5. Conclusions

The findings of this study add to the existing evidence that school gardening programmes are a promising and fun way for children to experience nature and promote vegetable intake. Understanding children’s perspectives facilitates tailoring existing or new programmes to the wishes and expectations of children, thereby increasing the extent to which the programme is enjoyed by children. This, in turn, may enhance children’s intrinsic motivation for gardening and vegetable consumption. To achieve greater vegetable consumption by children, efforts should not merely concentrate on how to motivate children to consume more vegetables, but on how to create supportive environments that enhance children’s intrinsic motivation to try new vegetables by satisfying their needs for autonomy, competence, and relatedness. Efforts could be made to create a better balance between structure and autonomy to choose, experiment and explore as health promotion programmes for children are often designed and implemented in a structured top-down manner. Furthermore, the feasibility and number of gardening assignments should be evaluated to ensure that they can be completed within the time available. This includes assessing whether and how children’s gardening time can be increased and the amount and duration of instructions decreased, and whether it is beneficial to provide children with additional instruction cards that facilitate remembering gardening assignments and enable them to work at their own pace. All of this requires the involvement of children in the development and evaluation of programmes. As the primary intervention users, children understand and experience what works and what does not, are able to identify shortcomings and provide valuable input for improvements. Additionally, children’s feelings of relatedness may be enhanced by involving parents in the programme, creating a link between school gardens and children’s homes. Finally, further research should evaluate whether improvements in gardening programmes are leading to higher, sustainable levels of vegetable intake. 

## Figures and Tables

**Table 1:** Participant demographics.

	*n*, Mean (%, SD)
Total	45
Gender, female (*N*, %)	27 (60.0)
Age, years (mean, SD)	9.5 (0.54)
Ethnicity (*N*, %)	
Dutch	27 (60)
Moroccan	5 (11.1)
Surinamese	2 (4.5)
Afghan	1 (2.2)
Mixed Dutch-immigrant descent	6 (13.3)
Mixed non-Dutch descent	1 (2.2)
Missing	3 (6.7)

*n*: number, %: percentages, SD: standard deviation.
